# Efficacy of topical carbonic anhydrase inhibitors in treating taxane drug-induced cystoid macular edema: A case report

**DOI:** 10.1097/MD.0000000000040958

**Published:** 2025-01-03

**Authors:** Xianbing Hou, Dandan Chen, Yingxue Lu

**Affiliations:** a Fenghua Hospital of Traditional Chinese Medicine, Ningbo, China.

**Keywords:** case report, cystoid macular edema, nab-paclitaxel, taxane

## Abstract

**Rationale:**

Taxanes, derived from Taxus chinesnsis, stabilize microtubules and include drugs like Paclitaxel, Docetaxel, and Nab-paclitaxel. These are commonly used to treat various malignant tumors. However, Taxane-drug-induced cystoid macular edema (TDICME) is a rare and often under-recognized complication.

**Patient concerns:**

A male patient, aged sixty-three, who was diagnosed with poorly differentiated gastric adenocarcinoma, experienced a progressive decline in visual acuity in both eyes after a 4-month course of nab-paclitaxel therapy.

**Diagnoses:**

Upon Fundus examination, bilateral cystoid macular edema (CME) was seen.

**Interventions:**

Undergo treatment with carbonic anhydrase inhibitors and discontinue the use of nab-paclitaxel.

**Outcomes:**

After eleven days of treatment with carbonic anhydrase inhibitors, the patient reported significant improvement in visual acuity. Furthermore, CME was completely resolved in both eyes 8 weeks after stopping nab-paclitaxel.

**Lessons:**

This case highlights the potential therapeutic effectiveness of topical carbonic anhydrase inhibitors in treating TDICME. Our findings underscore the importance of monitoring and addressing ocular side effects in patients undergoing Taxane therapy, ultimately contributing to enhanced patient quality of life and treatment outcomes.

## 1. Introduction

Tomography, case report Overview Taxanes are a group of substances that stabilize microtubules and are obtained from Taxus chinesnsis, often known as purple jackets. Taxanes, including Paclitaxel (Taxol®), Docetaxel (Taxotere®), and Nab-paclitaxel (Abraxane®), are often used by individuals for the treatment of several malignant tumors.^[1,2]^ Taxane-drug-induced cystoid macular edema (TDICME) is a recognized although infrequent consequence of treatment, as documented in the literature. TDICME imaging shows the presence of fluid on an ocular coherence tomography scan (OCT), but fundus fluorescein angiography shows little leakage. This study examines the efficacy of topical carbonic anhydrase inhibitors, namely 1% Brinzolamide eye drops, in the treatment of TDICME. Additionally, it explores the existing literature pertaining to this medical issue.

## 2. Case report

**Figure 1.**
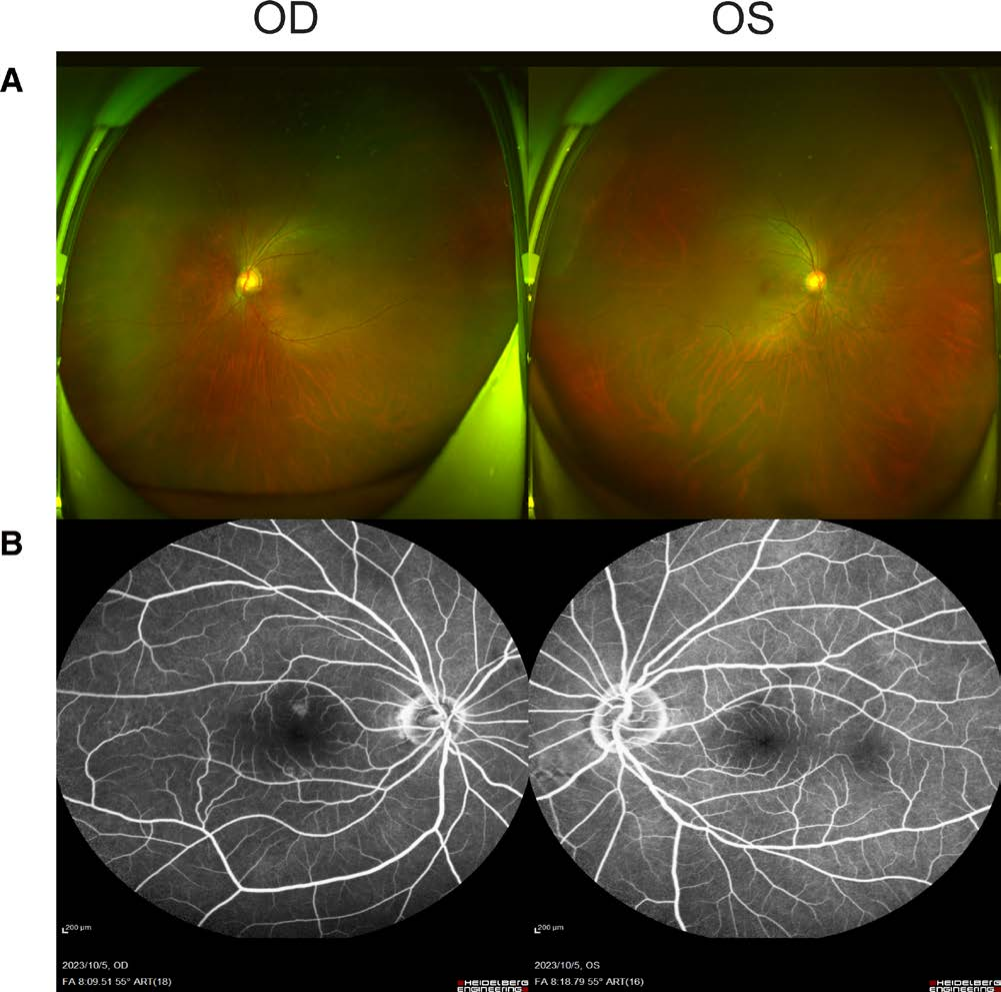
Fundus examination of both eyes at the initial consultation. Ultra-wide field fundus images showed macular edema in both eyes on first vision (A). The fluorescein angiograms showed litter leakage in right eye and no leakage in left eye on first vision (B).

**Figure 2.**
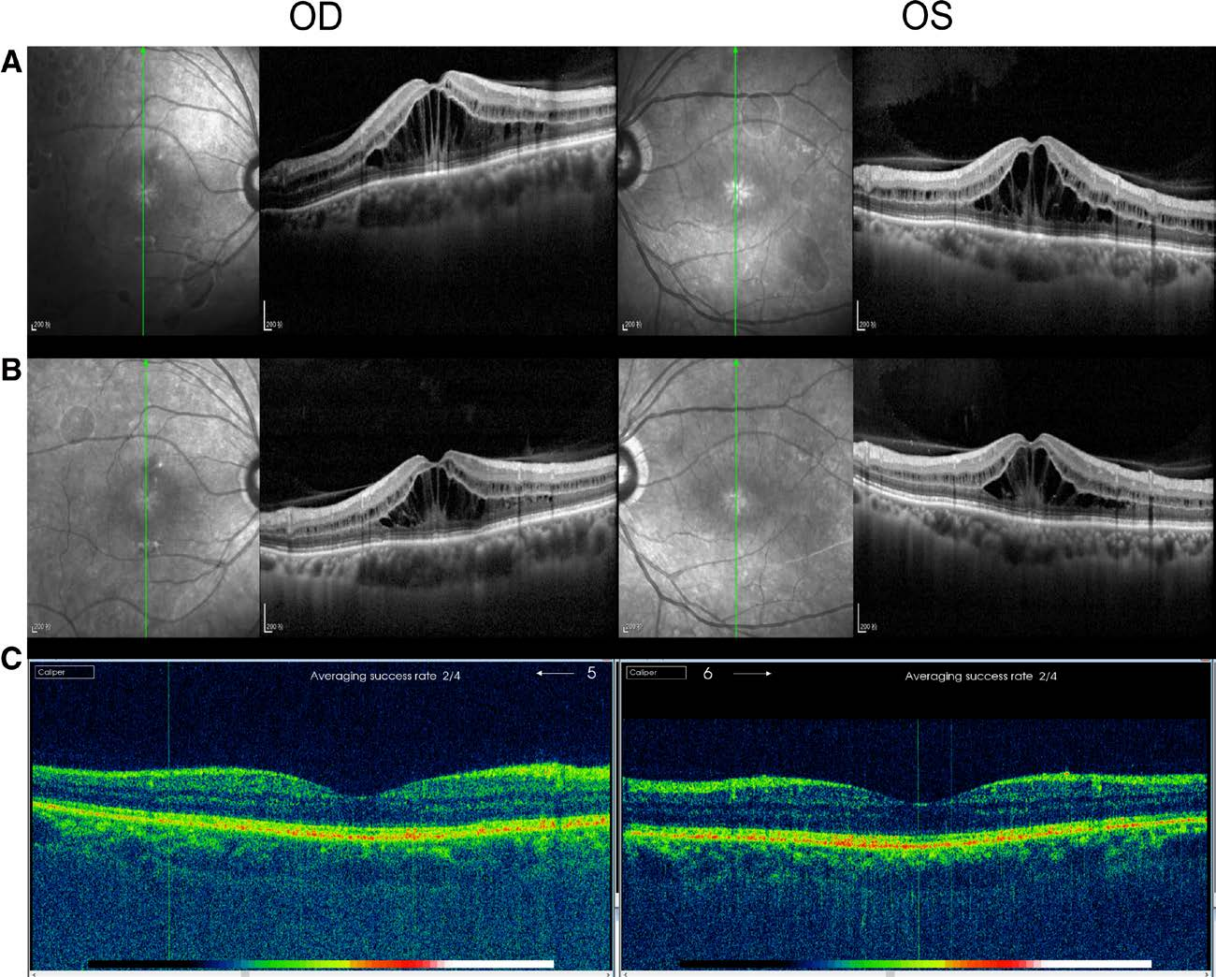
The change of macular edema. Optical coherence tomography scan showed the cystoid edema with a foveal thickness of 608 µm in the right eye and 612 µm in the left eye on first vision (A).Optical coherence tomography scan showed the cystoid edema with a foveal thickness of 552 µm in the right eye and 554 µm in the left eye after 4 weeks of discontinued of taxane (B). Optical coherence tomography scan showed the cystoid edema with a foveal thickness of 167 µm in the right eye and 173 µm in the left eye after 8 weeks of discontinued of taxane (C).

A 63-year-old man was diagnosed with poorly differentiated stomach adenocarcinoma 4 years ago. The patient had a comprehensive surgical procedure consisting of radical gastrectomy, esophagojejunostomy, and abdominal lymph node dissection. He began 8 rounds of treatment with docetaxel in June 2022. In May 2023, a total of 8 cycles of chemotherapy were started using the nab-paclitaxel 200 mg/dL, d8 regimen, resulting in a cumulative dosage of 3200 mg. The patient reported a progressive decline in visual acuity in both eyes over a period of 2 weeks, starting in September 2023. The first ophthalmologic examination revealed that the best-corrected visual acuity in the right eye was 0.4, while in the left eye it was 0.5. The evaluation of the anterior portion indicated normal findings. The intraocular pressures in both eyes were within the usual range. Upon Fundus examination, bilateral cystoid macular edema (CME) was seen (Fig. 1A). Figure 1B displays the results of fundus fluorescein angiography, indicating little leakage in the right eye and no leakage seen in the left eye. Verified with an ocular coherence tomography scan, the right eye had a central macular thickness of 608 μm, whereas the left eye had a central macular thickness of 612 μm (Fig. 2A). The degree of prominence of hyporeflective cysts was shown to be higher in the outer nuclear layer, but much lower in the inner nuclear layer. The patient has had excellent binocular vision from infancy, with no prior indications of eye illness or genetic eye disease in his family. He have not used eye medications for an extended period, have not used niacin medicines, and have no history of drug allergies. Subsequently, the diagnosis of taxane-drug-induced CME was established. The use of Nab-paclitaxel was terminated. We administered topical corticosteroids and nonsteroidal anti-inflammatory medications to the patient. Following a period of 4 weeks, his visual acuity had declined to 0.3 in the right eye and 0.2 in the left eye, accompanied with a little reduction in CME. The right eye had a central macular thickness of 552 μm, whereas the left eye had a central macular thickness of 554 μm (Fig. 2B). Due to the unresolved CME, we started his treatment with topical carbonic anhydrase inhibitors, namely 1% Brinzolamide eye drops administered twice daily. After a duration of eleven days after the use of carbonic anhydrase inhibitors, the patient reported a notable improvement in his visual acuity. Regrettably, the current state of the individual’s overall health has rendered it unfeasible to attend the scheduled ophthalmology follow-up. After a period of 8 weeks after the cessation of nab-paclitaxel, complete resolution of CME was seen in both eyes. The right eye had a central macular thickness of 167 μm, whereas the left eye had a central macular thickness of 173 μm (Fig. 2C). His visual acuity has enhanced to 0.5 in the right eye and 0.6 in the left eye.

The cessation of nab-paclitaxel and the administration of topical carbonic anhydrase inhibitors (Brinzolamide) resulted in significant improvement and eventual resolution of CME. Visual acuity improved substantially following the combined treatment regimen, underscoring the effectiveness of this therapeutic approach. This case highlights the importance of early detection and intervention in managing TDICME, emphasizing the potential benefits of brinzolamide in treating this condition. The patient’s case underscores the need for clinicians to monitor for ocular side effects in patients receiving taxane-based chemotherapy and to consider alternative treatments promptly when visual symptoms arise.

## 3. Literature review

We conducted a literature search on CME induced by albumin-bound paclitaxel, and as of March 22, 2024, we found 25 articles^[3–27]^ covering 27 cases (See Table 1 for details). Among these cases, there were 20 females and 7 males, with 3 cases reported domestically in China,^[18,20,25]^ and 4 cases reported in Japan,^[6,10,21]^ while the rest were from Europe and the United States. The cases included 17 with breast cancer, 2 with lung cancer, 7 with pancreatic cancer, and 1 with hypopharyngeal cancer. Except for 2 cases presenting unilaterally,^[6,16]^ CME induced by albumin-bound paclitaxel typically manifested bilaterally. The age of the patients ranged from 32 to 73 years, with the time interval between the initial use of albumin-bound paclitaxel and the onset of visual decline varying from 1.5 to 42 months. The shortest recovery time after discontinuation of the medication was 0.75 months, with the longest being 5 months. Patients treated with other drugs after discontinuing albumin-bound paclitaxel had an average recovery time of 2.15 months, compared to 2.81 months for those who did not use medication. This suggests that drug therapy may be effective for CME induced by albumin-bound paclitaxel.

**Table 1:** Case reports of cystoid macular edema due to albumin-bound paclitaxel

Author	Primary tumor	Sex	Age	Affected eye	Duration and dosage of treatment	Onset latency (months)	Treatment time to recovery (months)	Other treatments	Withdrawal
Smith et al^[3]^	Breast	FeMale	56	Bilateral	400 mg q3w (2.5 years)	28	3	No	Yes
Murphy, C. G^[4]^	Breast	FeMale	65	Bilateral	–	1.5	0.75	TS + NSAID	Yes
Murphy, C. G^[4]^	Breast	FeMale	58	Bilateral	11 months (dosage not specified)	11	3	No	Yes
Baskin et al^[5]^	Breast	FeMale	40	Bilateral	9 months (dosage not specified)	6	4	NSAID + GC	Yes
Yuko Tanaka^[6]^	Breast	FeMale	47	Left eye	400 mg q3w*7	4	2	No	Yes
Hassan T^[7]^	Breast	FeMale	73	Bilateral	3 months (dosage not specified)	3	4 months incomplete, lost	IVB	No
Ehlers et al^[8]^	Breast	FeMale	59	Bilateral	–	–	1	DRZ	Yes
Rahimy E^[9]^	Breast	FeMale	32	Bilateral	10 months (dosage not specified)	9	1.5	No	Yes
MATSUOKA N^[10]^	Breast	FeMale	39	Bilateral	400 mg q3w (1 year)	8	5 (OD), 6 (OS)	STTA (OD)	Yes
Rajesh C.Rao^[11]^	Breast	FeMale	45	Bilateral	–	–	–	No	Yes
Haider et al^[12]^	Lung	Male	73	Bilateral	4 years (dosage not specified)	42	2	No	Yes
Fenicia V^[13]^	Breast	FeMale	40	Bilateral	4 months (dosage not specified)	4	1	DEX + DRZ	-
Hassall et al^[14]^	Hypopharyngeal	Male	73	Bilateral	90 mg/m^2^ q4w*5	3	2	DRZ (OD),IVB (OS)	Yes
Park et al^[15]^	Breast	FeMale	69	Bilateral	6 months (100 mg/m^2^ d1, d8, d15)	6	2	TS	Yes
Ito er al^[16]^	Pancreas	FeMale	73	Left eye	4 months total 1031 mg	4	6	No	Yes
Lee et al^[17]^	Pancreas	FeMale	43	Bilateral	7 months (dosage not specified)	7	3	No	Yes
Xiaojia Song^[18]^	Lung	Male	53	Bilateral	250 mg/m^2^ d1, d8 q3w*6	5	–	–	–
Burgos-Blasco et al^[19]^	Pancreas	Male	67	Bilateral	6 months (125 mg/m^2^)	6	4	DEX	Yes
Ye, S et al^[20]^	Breast	FeMale	45	Bilateral	1.5 years total 9650 mg	18	2	IVR + DRZ	Yes
Mitsuru Otsubo et al^[21]^	Breast	FeMale	72	Bilateral	310 mg q3w*3	2	1.5 (OD),2.5 (OS)	DRZ	Yes
Mitsuru Otsubo et al^[22]^	Pancreas	Male	70	Bilateral	210 mg d1, d8, d15 q4w*5	5	1.25	DRZ	Yes
Ota et al^[23]^	Pancreas	Male	71	Bilateral	–	–	2	No	Yes
Alves, P.S et al^[24]^	Pancreas	Male	61	Bilateral	5 months (dosage not specified)	4	3	NSAID	Yes
M. Di Pippo et al^[25]^	Breast	FeMale	40	Bilateral	4 months (dosage not specified)	4	0.75	DEX (OD),DRZ (OS)	No
Jingwen Liu^[26]^	Breast	FeMale	60	Bilateral	400mg q3w*3	2.25	1	Oral Acetazolamide	Yes
Hiroaki Yamane et al^[27]^	Breast	FeMale	49	Bilateral	100 mg/m^2^ d1, d8, d15 q4w*7	19	2	STTA	Yes
Sridhar et al^[28]^	Pancreas	FeMale	48	Bilateral	–	–	–	No	No

DEX = dexamethasone, DRZ = dorzolamide, IVB = intravitreal bevacizumab injections, IVR = intravitreal ranibizumab injection, NSAID = nonsteroidal anti-inflammatory drug, STTA = sub-Tenon triamcinolone acetonide injection, TS = topical steroids.

## 4. Discussion

Paclitaxel (Taxol), docetaxel (Taxotere), and Nab-paclitaxel (Abraxane) of the taxane class are derived from the yew plant.^[1,2]^ These drugs function as inhibitors of microtubules, causing cytotoxicity by improving the assembly and stability of microtubules to hinder cellular division.^[28]^ Taxane medications are linked with an uncommon adverse effect called cystoid macular edema, often referred to as taxane drug-induced cystoid macular edema.

In individuals with TDICME, the occurrence of fluorescein leakage during fluorescein angiography is minimal or absent.^[15,29]^ TDICME often manifests in bilateral eyes, however, there have been documented instances of unilateral occurrences.^[30]^ These symptoms may emerge several months to 2.5 years after the injection of the medication.^[3,31]^ In their study, Alvarez et al^[32]^ examined fifty-seven instances documented in the literature, with an average occurrence of CME occurring around 4 months following the commencement of taxane therapy. The degree of prominence of hyporeflective cysts was shown to be higher in the outer nuclear layer, but much lower in the inner nuclear layer. In addition, the cysts are formed by fluid with a high viscosity. The CME pattern in the instances presented by Alvarez et al was clearly acknowledged. In their analysis, PeÁrez et al^[33]^ examine the distinctions between TDICME and CME, attributing them to other factors, including the existence of a continuous and unbroken outer plexiform layer, as well as an intact inner plexiform layer, on OCT. Furthermore, optical coherence tomography angiography (OCTA) did not detect any changes in the superficial and deep capillary plexus of the macular, nor did it detect any changes in the foveal avascular zone. The fluid in TDICME had a significant viscosity, resulting in the formation of a shadow underneath. The underlying mechanisms of TDICME are still not fully understood. One potential mechanism that may be considered is the impairment of Muller cells or the pumping function of RPE cells. The observed malfunction may be ascribed to the suppression of microtubule polymerization and intracellular transport, resulting in the buildup of intracellular fluid. Another alternate pathogenic theory is that the blood-retinal barrier is partially compromised, enabling the transport of tiny molecules rather than bigger ones (such as fluorescein).^[30,34,35]^ The lack of leakage seen on fundus angiography and indocyanine green angiography suggests that the observed fluid does not originate directly from the choroid. This may be attributed to the breakage of tight connections between retinal pigment epithelium (RPE) cells.^[36]^ The involvement of vascular endothelial growth factor in the pathogenetic pathway is not supported by the occurrence of CME upon intravenous injection of bevacizumab.^[37]^ Despite having completed 8 cycles of docetaxel prior to receiving albumin-bound paclitaxel, the patient did not exhibit any signs of macular cystoid edema. Nevertheless, the presence of macular cystoid edema after the administration of nab-paclitaxel indicates that there are diverse individual reactions to various taxanes. However, it is important to consider the potential for cumulative dosage effects between the 2 medications. The therapeutic method that is widely acknowledged in the medical community is the cessation of chemotherapy drugs, and it is seen that macular edema may be reversed with the cessation of drug administration.^[29]^ Nevertheless, the visual results might be influenced by the length of therapy and the duration of edema. Multiple writers have tried to use diverse interventions to expedite resolution or in cases when taxane medication cannot be terminated. Adjuvant therapy include a range of pharmaceutical interventions, such as carbonic anhydrase inhibitors, bevacizumab, corticosteroids, and nonsteroidal anti-inflammatory medications.^[19,34,38,39]^ The adjuvant therapies continue to be a subject of controversy. Carbonic anhydrase inhibitors have been shown to have a beneficial effect on improving visual acuity in the management of macular edema.^[14,30,35,37–40]^ Ehler et al^[8]^ conducted a monocular control study which demonstrated a faster correction of cystoid macular edema in the eye treated with a topical carbonic anhydrase inhibitor compared to the untreated eye when nab-paclitaxel was stopped. This paper proposes that topical carbonic anhydrase inhibitors may have potential therapeutic effectiveness in treating TDICME. Despite the cessation of Nab-paclitaxel, the use of topical carbonic anhydrase inhibitors may lead to a fast reduction in edema. Clinicians responsible for the supervision of patients undergoing taxane-based chemotherapy should exercise constant attention in detecting any changes in visual acuity. It is advisable for individuals to consider promptly transferring their condition to an ophthalmologist, since ceasing treatment may result in improvements in visual acuity.

While this case report suggests promising results with the use of topical carbonic anhydrase inhibitors for TDICME, several limitations must be acknowledged. Firstly, the study is based on a single patient case, which limits the generalizability of the findings. Further research involving a larger cohort of patients is necessary to validate these results. Additionally, the precise mechanisms underlying the efficacy of carbonic anhydrase inhibitors in resolving TDICME remain unclear and warrant further investigation. The lack of a control group in this observational study also limits the ability to draw definitive conclusions about the treatment’s efficacy. Finally, long-term follow-up is needed to determine the sustainability of the visual improvements observed and to assess any potential side effects associated with prolonged use of carbonic anhydrase inhibitors.

## Author contributions

**Conceptualization:** Yingxue Lu.

**Data curation:** Xianbing Hou, Dandan Chen, Yingxue Lu.

**Validation:** Dandan Chen.

**Visualization:** Xianbing Hou.

**Writing – original draft:** Yingxue Lu.

**Writing – review & editing:** Xianbing Hou, Yingxue Lu.
